# P05 The current evidence for transitional care in young people with chronic pain: a systematic review

**DOI:** 10.1093/rap/rkac067.005

**Published:** 2022-09-28

**Authors:** Lauren Huckerby, Janet E McDonagh, Rebecca R Lee

**Affiliations:** Royal Manchester Children's Hospital, Manchester University Hospitals Trust, Manchester, United Kingdom; Royal Manchester Children's Hospital, Manchester University Hospitals Trust, Manchester, United Kingdom; Centre for Epidemiology Versus Arthritis, Centre of Musculoskeletal Research, Division of Musculoskeletal and Dermatological Sciences, Faculty of Biology, Medicine and Health, University of Manchester, Manchester Academic Health Science Centre, Manchester, United Kingdom; National Institute of Health Research Biomedical Research Centre, Manchester University Hospital NHS Trust, Manchester, United Kingdom; Centre for Epidemiology Versus Arthritis, Centre of Musculoskeletal Research, Division of Musculoskeletal and Dermatological Sciences, Faculty of Biology, Medicine and Health, University of Manchester, Manchester Academic Health Science Centre, Manchester, United Kingdom; National Institute of Health Research Biomedical Research Centre, Manchester University Hospital NHS Trust, Manchester, United Kingdom

## Abstract

**Introduction/Background:**

Paediatric chronic musculoskeletal pain presents a significant individual and societal burden, with an estimated prevalence of 11-38%. A large proportion of young people (YP) with chronic pain are likely to have unresolved pain which continues into adulthood and as a result require transitional care.

To date, although there is guidance and transitional care research in a range of health conditions, there is limited evidence identifying the extent to which transitional care for YP with chronic pain has been developed or investigated in research.

Our objective was to review the current evidence for transitional care in young people with chronic pain.

**Description/Method:**

The PEO tool was used to develop a search strategy, where the Population was “young people”, the Exposure was “chronic pain” and the Outcome was “Transition to Adult Care” or variations of such words. Studies were identified by searching 4 databases: EMBASE, Medline, CINAHL and PsycINFO.

Inclusion criteria were: sample population age between 10-24 years, a confirmed diagnosis of a condition characterised by chronic pain, any health care setting, any service provider, published peer reviewed, English language. Excluded were case reports, editorials, abstracts, meta-analyses, books or book chapters. Searches took place between September and December 2021.

**Discussion/Results:**

98 papers were identified by the search; 14 were selected after abstract screening. Two independent reviewers then screened papers for inclusion, extracted data, and assessed the quality of the studies followed by a senior reviewer. Of the 14 papers, full text review found that none of the papers looked specifically at the evidence with respect to transitional care for YP with chronic pain. Of those which did not meet the inclusion criteria, there were 4 papers which highlighted the importance of considering transitional care for YP with chronic pain and informed our discussion.

**Key learning points/Conclusion:**

The lack of studies addressing transitional care for YP with chronic pain was surprising in view of the prevalence of chronic pain during adolescence and the reported importance to YP. To date, most research has considered disease-specific transitional care. Due to the lack of results reported here, the question remains as to whether transitional care for YP with chronic pain differs significantly from transitional care for other long-term health conditions. Unique challenges faced by YP with chronic pain have been proposed in the literature. However, we would argue that these are also experienced by YP with rheumatic conditions, for example, stigma and lack of belief of the condition. Consideration of whether transitional care is truly different for these YP is a useful starting point for future research.

Building on existing research will be important. For example, meeting the adult provider has been identified as a predictor of successful transition but is challenging when chronic pain is the primary condition, as there isn’t a clear pathway of which adult service these YP will transfer to. Likewise, appropriate parental involvement has been identified as a predictor of successful transition. Evidence has shown that parents of YP with chronic pain may restrict their child’s independence, and therefore will need to be addressed as part of transitional care provision.

Finally, chronic pain is a feature of many long-term health conditions, for example, sickle cell disease, cystic fibrosis, cerebral palsy. How pain management is addressed in existing transitional care provision for rheumatic conditions such as JIA and SLE and the relationship of pain to outcomes of transitional care needs further research. If effective interventions can be provided during these crucial years, the trajectory of these YP as adults can potentially be improved in the long-term, reducing the individual and societal burden of pain.

